# Impact of Plant Growth-Promoting Microorganism (PGPM) Consortium on Biochemical Properties and Yields of Tomato Under Drought Stress

**DOI:** 10.3390/life14101333

**Published:** 2024-10-18

**Authors:** Ram Krishna, Waquar Akhter Ansari, Mohammad Altaf, Durgesh Kumar Jaiswal, Sudhakar Pandey, Achuit Kumar Singh, Sudhir Kumar, Jay Prakash Verma

**Affiliations:** 1 ICAR—Indian Institute of Vegetable Research, Jakhini, Varanasi 221305, Uttar Pradesh, India; mbt.r.krishna@gmail.com (R.K.); waquar.ansari@gmail.com (W.A.A.); sudhakariivr@gmail.com (S.P.); sudhir2203@gmail.com (S.K.); 2 Institute of Environment and Sustainable Development, Banaras Hindu University, Varanasi 221005, Uttar Pradesh, India; 3 Marwadi University Research Centre, Marwadi University, Morbi Road, Rajkot 360003, Gujarat, India; 4 Department of Chemistry, College of Science, King Saud University, P.O. Box 2455, Riyadh 11451, Saudi Arabia; maltaf@ksu.edu.sa; 5 Department of Biotechnology, Graphic Era (Deemed to be University), Dehradun 248002, Uttarakhand, India; durgesh.jaiswal9@gmail.com

**Keywords:** tomato, drought, PGPM, growth attributes, soil physico-biological properties

## Abstract

Drought is the most important abiotic stress that restricts the genetically predetermined yield potential of the crops. In the present study, four tomato varieties: Kashi Vishesh, Kashi Aman, Kashi Abhiman, and Kashi Amrit, were used to study the effect of PGPMs (plant growth-promoting microorganisms). PGPM strains, *Bacillus megaterium* BHUPSB14, *Pseudomonas fluorescens* BHUPSB06, *Pseudomonas aeruginosa* BHUPSB01, *Pseudomonas putida* BHUPSB0, *Paenibacillus polymixa* BHUPSB17, and *Trichoderma horzianum*, were used as the consortium. The control group was irrigated up to 80% of field capacity, while 7-, 14-, and 21-day water-deficit-exposed (DWD) plants’ pot soil moisture was maintained to 40, 25, and 15% of the field capacity, both with and without the PGPM inoculation condition. The physiological parameters, such as electrolyte leakage, relative water content, photosynthetic efficiency, and chlorophyll color index, were significantly improved by PGPM application under progressive drought stress, compared to the control. PGPM application enhanced the proline accumulation and reduced the formation of hydrogen peroxide and lipid peroxidation under drought stress. The plant growth attributes were significantly increased by PGPM application. The Kashi Amrit variety showed the highest fruit yield among the four varieties under all the treatments. The PGPM consortium application also improved the soil physico-biological properties and nutrient availability in the soil. The PGPM consortium used in this study can potentially mitigate drought stress on tomato in drought-prone regions and act as a biofertilizer. The present study will open a new avenue of drought stress management in tomato.

## 1. Introduction

Tomato (*Solanum lycopersicum* L.) is the second most important vegetable crop in the *Solanaceae* family. It is characterized by low calorie values and presents a rich source of minerals, proteins, vitamins, and bioactive compounds, with many health benefits [1,2]. Presently, about 186 million tons of tomatoes is produced on 4.9 million hectares globally. China is the largest producer, followed by India and the United States, which produce 68.34, 20.69, and 10.19 million tons, respectively (https://www.fao.org/faostat/en/#data/QCL, accessed on 15 July 2024). Under natural conditions, the production and quality of tomato is hampered by abiotic stresses, such as drought, high and low temperatures, and water logging [3,4,5]. Drought is a meteorological condition that indicates the water deficit due to low water availability [6]. Drought stress induces changes in plants on morphological, physiological, biochemical, and molecular levels [7], and disturbs normal plant growth and development [8]. Additionally, it affects the availability of NO_3_^−^, SO_3_^−2^, Ca, Mg, and Si, as well as other soil nutrients [9], and generates free radicals, such as superoxide, hydroxyl, and hydrogen peroxide, which disturb plants’ defense systems [8]. Drought stress causes up to 70% yield loss depending on the growth stage of the plant [2]. In the last decades, several breeding and transgenic approaches for tomato aimed to develop drought tolerance, but they saw very limited success. This can be ascribed to drought-tolerant gene/s, hurdles in gene transfer, and acceptance of modified plants due to social and ethical issues [10]. Plant growth-promoting microorganisms (PGPMs) are emerging as an eco-friendly tool for crops’ abiotic stress tolerance by improving plant and soil health [11]. Many reports suggests that damage might be ameliorated by applying various PGPM inocula [12]. The microbes colonize the rhizosphere, and directly or indirectly impact plant tolerance against abiotic stress. The major modes of action are production of phytohormones, such as abscisic acid (ABA), cytokinin, and indole-3-acetic acid (IAA), exopolysaccharides’ secretion, ACC deaminase, and tolerance mediated through induced systemic resistance. *Pseudomonas putida*, *Bacillus megaterium*, *Pseudomonas aeruginosa*, *Paenibacillus polymyxa*, and *Pseudomonas fluorescens* strains improve drought stress tolerance in plants by maintaining stomatal conductance, photosynthetic pigments, relative water content, increased osmoprotectants, increased lengths of the shoots and yields of biomass, and other different traits [13,14,15,16].

Furthermore, certain PGPMs, such as *Pseudomonas mendocina*, *Glomus intraradices*, *Pseudomonas aeruginosa*, *Trichoderma harzianum*, *Pseudomonas fluorescens*, and various Pseudomonas species, establish symbiotic relationships with plants, colonizing their roots. This interaction triggers the upregulation of stress-responsive genes. Increased expression of genes in plants exposed to a blend of endophytic and rhizospheric PGPMs enhances stress resilience by facilitating antioxidative enzymes to scavenge reactive oxygen species (ROS) [17]. Similar to other vegetables, tomatoes are highly vulnerable to drought stress and have been extensively researched to comprehend the impacts of drought stress and explore potential strategies to mitigate the losses incurred [18]. In the past, PGPMs, such as *Trichoderma harzianum*, *Pseudomonas aeruginosa*, *Pseudomonas putida*, *Pseudomonas fluorescens*, *Bacillus megaterium*, and *Paenibacillus polymixa*, have been utilized individually or in various combinations to alleviate drought stress in crops, including tomato, mung bean, sunflower, pea, maize, *Lactuca sativa*, and *Arabidopsis* [16,17,19]. However, it is hypothesized that combining these PGPMs into a single consortium might offer superior drought alleviation properties compared to individual application or combinations of just one or two PGPMs. This study aims to investigate the effects of six PGPMs: *Bacillus megaterium* BHUPSB14, *Pseudomonas fluorescens* BHUPSB06, *Pseudomonas aeruginosa* BHUPSB01, *Pseudomonas putida* BHUPSB0, *Paenibacillus polymixa* BHUPSB17, and *Trichoderma harzianum*, on four tomato varieties subjected to controlled water-deficit irrigation.

## 2. Material and Methods

### 2.1. PGPM Strains

The pure culture of six PGPM strains: *Bacillus megaterium* BHUPSB14, *Pseudomonas fluorescens* BHUPSB06, *Pseudomonas aeruginosa* BHUPSB01, *Pseudomonas putida* BHUPSB0, *Paenibacillus polymixa* BHUPSB17, and *Trichoderma harzianum*, were collected from Banaras Hindu University, Varanasi, India.

### 2.2. Tomato Variety, PGPM Inoculation, and Experimental Setup

In the present study, four tomato varieties suitable for cultivation in Uttar Pradesh: Kashi Vishesh, Kashi Aman, Kashi Abhiman, and Kashi Amrit, developed by ICAR—Indian Institute of Vegetable Research, were selected for the experiment. The study was carried out in an insect-proof screen house. The individually grown PGPM strains: *Bacillus megaterium* BHUPSB14, *Pseudomonas fluorescens* BHUPSB06, *Pseudomonas aeruginosa* BHUPSB01, *Pseudomonas putida* BHUPSB0, *Paenibacillus polymixa* BHUPSB17, and *Trichoderma horzianum*, were mixed to develop the consortium. Seedlings aged 25 days old were treated with a 10% sucrose solution for 5 min and then inoculated with the consortium for 25–30 min. They were subsequently transplanted into 7 kg pots and organized into 4 sets, each containing 9 plants. In the control group, soil moisture was maintained at 80% of field capacity. For the drought treatments, soil moisture was maintained at 40%, 25%, and 15% of field capacity for 7, 14, and 21 days of water-deficit (DWD) treatment, respectively, with or without PGPM inoculation. The top-four fully expanded leaf samples were collected from 7, 14, and 21 DWD-treated plants, along with the 0-day controls, for physiological and biochemical analysis. For every harvesting, three replicates were collected.

### 2.3. Relative Water Content (RWC) and Electrolyte Leakage (EL)

To measure the RWC and EL, we followed the well-established protocol of Khare et al. [20]. Initially, fresh leaf samples were taken and weighed (fresh weight (FW)), dipped in water for 6 h until they became fully turgid, and then weighed again (turgid weight (TW)). Then, they were kept for 24 h at 80 °C in an oven and weighed again (dry weight (DW)). To measure the RWC, the following formula was followed: RWC% = (FW − DW/TW − DW) × 100. To measure the EL, we used a conductivity meter (CM-180, Elico, India). For this measurement (EC1), 8 leaf discs of 1 cm in size were kept for 4 h in 25 mL of distilled water at room temperature. Consequently, they were autoclaved at 121 °C for 30 min, then EC2 was measured, and EL was calculated using the following formula: EL% = (EC1/EC2) × 100.

### 2.4. F_v_/F_m_ and Chlorophyll Color Index (CCI)

The photosynthetic efficiency of plants was recorded in terms of *F_v_*/*F_m_* using the Handy Plant Efficiency Analyzer (Hansatech Instruments, King’s Lynn, Norfolk, UK). For 30 min, dark adaptation clips were placed on an adaxial surface. Excitation irradiance was set at 3000 μM/m^2^/s^1^. Both the minimum (*F*_0_) and maximum (*F_m_*) chlorophyll fluorescence were measured, and the photosystem II was calculated using the following formula: *F_v_*/*F_m_* = *F_m_* − *F*_0_/*F_m_*. The first, second, and third leaves from the apex were used for CCI measurements during the day time, 10.00–11.00 a.m., using the Portable Chlorophyll Meter CCM-200 (Opti-Sciences, Tyngsboro, MA, USA) at 655/940 nm.For this measurement, five plants were selected randomly [21].

### 2.5. Hydrogen Peroxide (H_2_O_2_), Lipid Peroxidation (MDA), and Proline Quantification

Hydrogen peroxide was measured as per the procedure of Shah et al. [22]. Leaf samples (0.2 g) were homogenized in 50 mM phosphate buffer (pH 6.5), centrifuged at 7500× *g* for 15 min, and the supernatant was mixed with 1 mL of 0.1% titanium sulphate. Finally, the mixture was centrifuged for 15 min at 7000× *g*, and the intensity was measured using a UV-Vis spectrophotometer at 410 nm (Perkin Elmer 2380, Billerica, MA, USA). Lipid peroxidation in terms of malondialdehyde (MDA) was measured as per the protocol of Heath and Packer [23]. Leaf tissue samples of 0.3 g were homogenized in 3 mL of a 0.1% (*w*/*v*) solution of trichloroacetic acid (TCA). The mixture was centrifuged at 10,000× *g* for 20 min, and 0.5% thiobarbituric acid (TBA) in 20% TCA was added to 2 mL of supernatant, then kept for 30 min at 95 °C. The reaction was rapidly cooled on ice and later centrifuged for 10 min at 10,000× *g*. The absorbance of the resultant supernatant was measured using a UV-Vis spectrophotometer (Perkin Elmer 2380, Billerica, MA, USA) at 532 and 600 nm. Measurements of leaf proline contents were performed as per the procedure of Bates et al. [24]. Fresh leaf tissue samples of 0.5× *g* were homogenized in sulfosalicylic acid and centrifuged. To 2 mL of the supernatant, ninhydrin and acetic acid were added and incubated at 100 °C for 1 h. Further extraction was performed by adding 4 mL of toluene for 15–20 s. The absorbance was recorded at 520 nm using a UV-Vis spectrophotometer (Perkin Elmer 2380, Billerica, MA, USA).

### 2.6. Morphological Parameters

To measure the shoot weight, three plants were carefully removed from the pots, washed, and their surfaces were dried. The plants were then divided into shoot and root portions, with shoot weight recorded in grams and root length measured in centimeters using a balance and a meter tape, respectively. Additionally, the fruit count for each plant was conducted, and the total fruit weight per plant was recorded.

### 2.7. Physiochemical and Nutritional Properties of Soil

Different physiochemical parameters of the soil were measured using well-established protocols. Following Jackson’s methodology, soil electrical conductivity was measured using a conductivity meter [25], while soil pH was determined using a pH meter according to the protocol established by Yan et al. [26]. K_2_Cr_2_O_7_ oxidation methods, as presented by Salam et al. [27], were used to measure the soil organic carbon (OC). In addition to this, for the measurements of available N, P, and K, we implemented the protocols presented by Subbiah and Asija [28], Olsen et al. [29], and Kirkbright and Aargent [30].

### 2.8. Soil Microbial Population

Soil microbial populations were quantified using serial dilution and plating methods [31]. For the serial dilution, 1 g of rhizospheric soil was mixed with 9 mL of sterile 0.85% saline water in a test tube, designated as the first dilution (10⁻^1^). Subsequent dilutions up to 10⁻⁶ were prepared by adding 0.1 mL aliquots from each dilution to the corresponding test tubes. From each dilution, 100 μL aliquots were transferred and spread aseptically on a petri plate containing media, such as Nutrient agar, Kenknight and Munaier’s agar, and Potato Dextrose Agar for selective growth of bacteria, actinomycetes, and fungal populations, respectively. Later, plates were incubated at 30 °C for 2–5 days. After completion of incubation, plates were inspected in different dilutions, and the microbial colony counts were performed and expressed as total colony-forming units (CFUs).

### 2.9. Soil Dehydrogenase Activity

Dehydrogenase activity was assessed as per the methodology of Casida et al. [32]. The soil sample (5 g) was placed in an Erlenmeyer flask (50 mL), and 0.1 g of CaCO_3_ was added, followed by the addition of 1 mL of 3% triphenyltetrazolium chloride (TTC) and 4 mL of distilled water. Through gentle tapping, the suspension was mixed, and then incubated at 37 °C for 24 h. After incubation, 40 mL of acetone was added to the extraction and filtered. The pink color that developed then disappeared after adding excess acetone, and the final volume was adjusted to 50 mL with acetone. The absorbance was measured at 485 nm using a spectrophotometer (Model 2202, Systronics, Ahmedabad, Gujarat, India) to assess triphenylformazan (TPF) in the samples.

### 2.10. Soil Urease Activity

Kandeler and Gerber’s methods were implemented to measure the urease activity [33]. An accurately weighed, moist soil sample (5 g) was placed in an Erlenmeyer flask, to which urea solution (2.5 mL) was added. The flask was then incubated at 37 °C for 2 h; then, 50 mL of KCl solution was added and shaken for 30 min. The resultant filtrate was used to measure the ammonium content on a UV-Vis spectrophotometer (Model 2202, Systronics, India) at 690 nm. For ammonium determination, 1 mL of the clean filtrate was poured into an Erlenmeyer flask (50 mL), supplemented with double-distilled water (9 mL), Na salicylate/NaOH solution (5 mL), and sodium dichloro-isocyanurate solution (2 mL). Absorbance was measured at 690 nm.

### 2.11. Soil Phosphatase Activity

Phosphatase activity was assessed according to the procedure of Eivazi and Tabatabai [34]. For determination of phosphatase activity, a soil sample (1 g) was placed into an Erlenmeyer flask (50 mL) and treated with 0.25 mL of toluene, 4 mL of modified universal buffer (MUB; pH 11 for the assay of alkaline phosphatase), and 1 mL of p-nitrophenyl phosphate solution, made in the same buffer. Flasks were closed and mixed, then incubated for 1 h at 37 °C. After the incubation, 1 mL of CaCl_2_ (0.5 M) and 4 mL of NaOH (0.5 M) were added and mixed. The absorbance was measured at 400 nm.

### 2.12. Statistical Interpretation

Statistical analysis was performed using SPSS software (Version 16.0, SPSS Inc., Chicago, IL, USA). Each experiment comprised three independent biological replicates, each conducted in triplicate. Statistical analysis utilized the mean value of each replication, with significance differences assessed via one-way analysis of variance (ANOVA). Mean separations were compared using Duncan’s multiple-range test at a significance level of *p* ≤ 0.05. The correlation analysis was performed using the PAST version 4.03 software.

## 3. Results

### 3.1. RWC and EL

**Figure 1:**
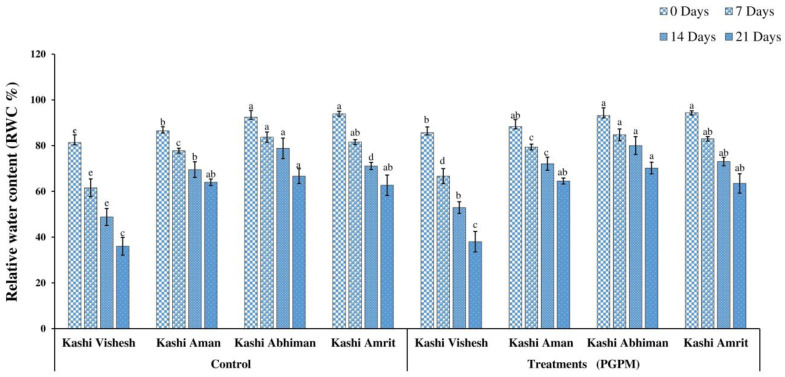
Effect of the PGPM consortium on the relative water content (RWC) in leaves of different tomato varieties under drought stress. All the values are means of three replicates ± SD. ANOVA was found significant at the 95% significance level. Different symbols indicate significantly different values under both control and treatment (PGPM) conditions for a particular drought (i.e., 0, 7, 14, and 21 DWD) treatment (DMRT ≤ 0.05).

**Figure 2:**
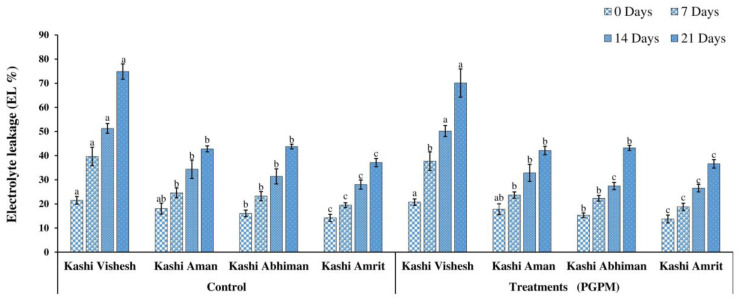
Effect of the PGPM consortium on electrolyte leakage (EL) in leaves of different tomato varieties under drought stress. Details are mentioned in Figure 1.

The relative water content (RWC) decreased with the prolongation of the water deficit, and the maximum reduction was detected after 21 DWD in all varieties. The RWC of all the varieties was significantly improved with the PGPM treatment. The maximum reduction recorded in Kashi Vishesh was 36.03% without PGPM after 21 DWD, while it was 70.19% in the case of Kashi Abhiman with PGPM (Figure 1). EL increased after 7, 14, and 21 DWD in both non-treated and treated plants. The order of EL under the control condition, without PGPM treatment, was as follows: Kashi Vishesh > Kashi Aman > Kashi Abhiman> Kashi Amrit, and a similar order was observed in PGPM-treated plants. EL recorded a maximum after 21 DWD in Kashi Vishesh (51.29%) and a minimum in Kashi Amrit (28.01%), and the order for non-treated plants was as follows: Kashi Vishesh > Kashi Abhiman> Kashi Aman > Kashi Amrit. A similar order was recorded under PGPM-treated conditions (Figure 2).

### 3.2. F_v_/F_m_ and CCI

**Figure 3:**
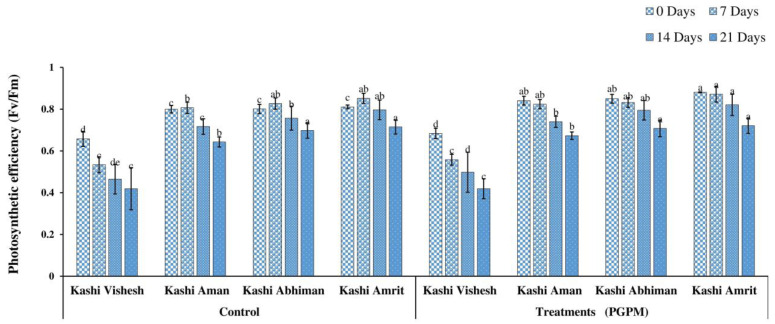
Effect of the PGPM consortium on *F_v_*/*F_m_* in leaves of different tomato varieties under drought stress. Details are mentioned in Figure 1.

**Figure 4:**
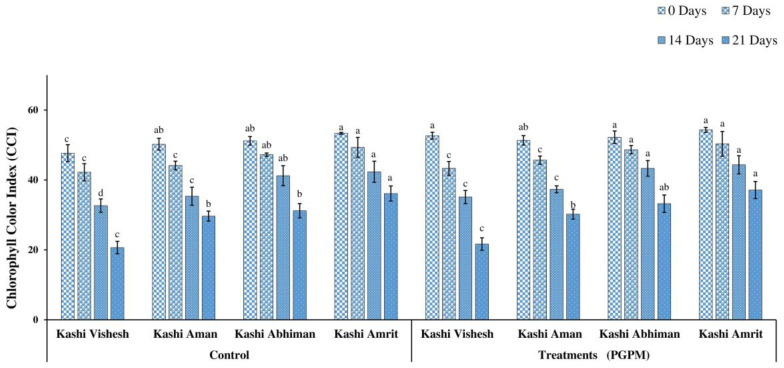
Effect of the PGPM consortium on CCI in leaves of different tomato varieties under drought. Details are mentioned in Figure 1.

F_v_/F_m_ reduced with the increase in DWD, with the maximum value (0.850) under the control condition and the minimum (0.419) value after 21 DWD. The trend of control plants was Kashi Amrit > Kashi Abhiman > Kashi Aman > Kashi Vishesh, while the trend for PGPM-treated plants was Kashi Amrit > Kashi Abhiman > Kashi Aman > Kashi Vishesh. However, after 21 DWD, similar to the trend noted for the control plants, the same trends were also recorded for PGPM-treated plants (Figure 3). Similar to *F_v_/F_m_*, reductions in CCI were also recorded, and the reduction was maximum after 21 DWD. Under the control condition, the maximum CCI was recorded in Kashi Amrit (53.33), while it was lowest in Kashi Vishesh (47.66). The PGPM treatment significantly improved the CCI: it was minimum in Kashi Aman (51.33) and maximum in Kashi Amrit (54.33). Although, after 21 DWD, it was 20.66 (Kashi Vishesh) and 36.12 (Kashi Amrit) in control plants, while it was 21.66 (Kashi Vishesh) and 37.11(Kashi Amrit) in PGPM-treated plants (Figure 4).

### 3.3. Hydrogen Peroxide (H_2_O_2_) and Lipid Peroxidation (LPO)

**Figure 5:**
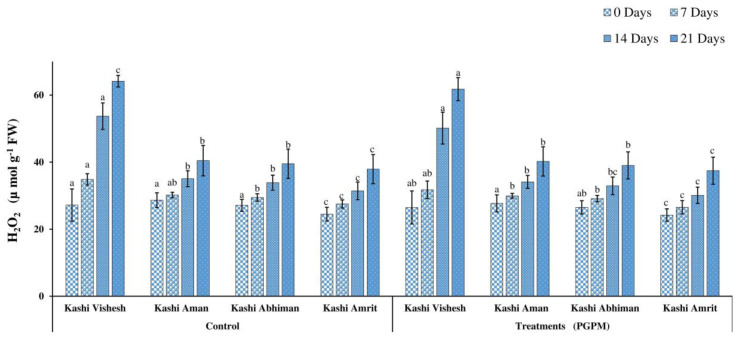
Effect of the PGPM consortium on hydrogen peroxide (H_2_O_2_) in different tomato varieties under drought stress conditions. Details are mentioned in Figure 1.

**Figure 6:**
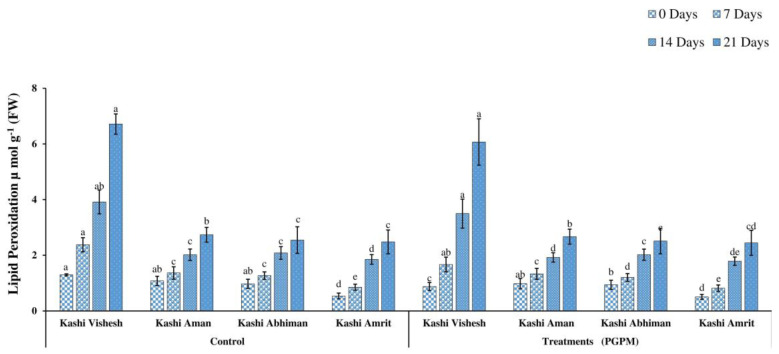
Effect of the PGPM consortium on lipid peroxidation (LPO) in different tomato varieties under drought conditions. Details are mentioned in Figure 1.

The H_2_O_2_ ranged from 24.23 to 64.16 (µ mol g^−1^ (FW)) and increased after 7, 14, and 21 DWD in both control and PGPM treated Kashi Vishesh, Kashi Aman, Kashi Abhiman, and Kashi Amrit plants. H_2_O_2_ was at a minimum of 24.23 (µ mol g^−1^ (FW)) in Kashi Amrit under the control and PGPM-treated conditions, while it was at a maximum of 28.66 and 27.71 (µ mol g^−1^ (FW)) for Kashi Aman. The highest H_2_O_2_ was recorded at 64.16 and 61.78 (µ mol g^−1^ (FW)) for Kashi Vishesh under the control and PGPM-treated conditions (Figure 5). The LPO concentration increased from 0.538 to 3.916 (µ mol g^−1^ (FW), after 7, 14, and 21 DWD in Kashi Vishesh, Kashi Aman, Kashi Abhiman, and Kashi Amrit plants under the control and PGPM-treated conditions. Under the control condition, the maximum LPO (1.296 µ mol g^−1^ (FW)) was noted in Kashi Vishesh (control), and the minimum (0.503 µ mol g^−1^ (FW)) in Kashi Amrit with PGPM treatment (Figure 6).

### 3.4. Proline Content

**Figure 7:**
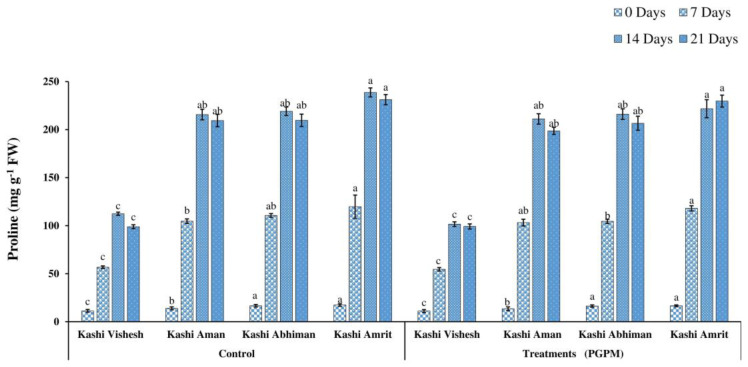
Effect of the PGPM consortium on proline content in different tomato varieties under drought conditions. Details are mentioned in Figure 1.

In both the control and PGPM-treated conditions in Kashi Vishesh, Kashi Aman, Kashi Abhiman, and Kashi Amrit, the proline level increased (11.29 to 118.1 mg g^−1^ FW) up to 7 and 14 DWD, and decreased after 21 DWD. Under the control condition, the maximum proline level noted in Kashi Amrit control plants was 17.34 mg g^−1^ FW, while a minimum level of 11.11 mg g^−1^ FW was noted in Kashi Visheshcontrol plants. Although, a maximum proline level of 238.7 mg g^−1^ FW was recorded in Kashi Amrit control plants after 14 DWD (Figure 7).

### 3.5. Total Fruit Number and Fruit Yield

**Figure 8:**
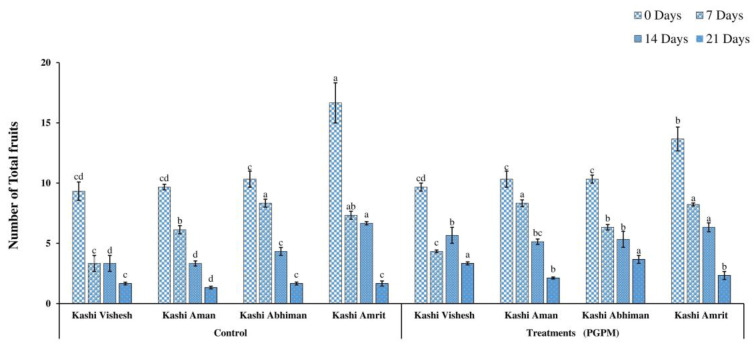
Effect of the PGPM consortium on the number of total fruits in different tomato varieties under drought conditions. Details are mentioned in Figure 1.

**Figure 9:**
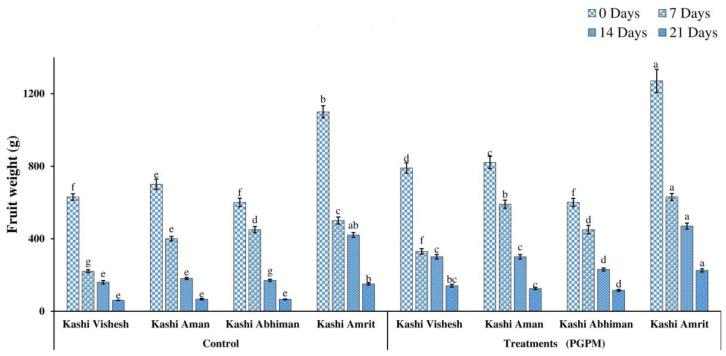
Effect of the PGPM consortium on fruit weights in different varieties of tomato under drought conditions. Details are mentioned in Figure 1.

Reductions in the numbers of fruit (16.66–1.33) and the fruit weights (1100–60.33 g) were recorded after 7, 14, and 21 DWD in all varieties under control and PGPM-treated conditions. Under 0 DWD in control plants, the maximum number of fruits (16.66) was recorded in Kashi Amrit and the minimum in Kashi Vishesh (9.33). However, the maximum fruit weight of 1270 g was recorded in Kashi Amrit PGPM-treated plants, and the minimum of 600.6 g in Kashi AbhimanPGPM-treated plants. Although, after 21 DWD, fruit number was the highest (3.66) in Kashi Abhiman PGPM-treated plants and the lowest (1.333) in Kashi Aman control plants (Figure 8). Similarly, after 21 DWD, the maximum fruit weight (225.3 g) was recorded in Kashi Amrit PGPM-treated plants, and the minimum fruit weight-(60.33 g) was recorded in Kashi Vishesh control plants (Figure 9).

### 3.6. Soil pH, Electrical Conductance (EC), and Organic Carbon (OC)

Under 0, 7, 14, and 21 DWD, the pH values of the soil were measured, with a non-significant difference noted in Kashi Vishesh, Kashi Aman, Kashi Abhiman, and Kashi Amrit. Similarly, non-significant differences were recorded when the plants were treated with PGPMs (Table 1). An increase (0.448–0.512 dSm^−1^) in electrical conductivity (EC) of the soils of Kashi Vishesh, Kashi Aman, Kashi Abhiman, and Kashi Amrit plants was noted with the increase in DWD; however, the increase was not significant under different water treatments, i.e., at 0, 7, 14, and 21 DWD. Similarly, when Kashi Vishesh, Kashi Aman, Kashi Abhiman, and Kashi Amrit plants were treated with PGPMs, an increase in electrical conductivity under 7, 14, and 21 DWD was noted; however, higher electrical conductivity (0.480–0.553 dSm^−1^) was noted compared to the plants under the control condition (Table 2). The organic content (OC) of Kashi Vishesh, Kashi Aman, Kashi Abhiman, and Kashi Amrit plants’ soil decreased with the increase in DWD. It was a maximum of 0.621% in Kashi Aman under 0 DWD and a minimum of 0.455% in Kashi Vishesh under 21 DWD, in both control plants and those under PGPM treatment. In the case of control plants, the maximum OC under 0 DWD was noted in the Kashi Aman (0.621%) plant’s soil, while it was lowest under 21 DWD in the case of the Kashi Vishesh (0.455%) plant’s soil. In the PGPM-treated plants, the maximum OC % was noted in the Kashi Abhiman (0.669%) plant’s soil under 0 DWD, and the minimum value was recorded in the Kashi Amrit (0.517%) plant’s soil after 21 DWD (Table 3).

### 3.7. Microbial Population

A reduction in the microbial population under control and PGPM treatment conditions was noted: the bacterial population ranged from 23.81 to 15.21 × 10^4^ CFU g^−1^ in the control and 24.63 to 19.57 CFU g^−1^ with PGPM treatment under 0 to 21 DWD. The PGPM treatment significantly improved the bacterial population under different DWDs. The maximum bacterial population was recorded in the Kashi Abhiman (23.81 × 10^4^ CFU g^−1^) controland in the Kashi Amrit (24.63 × 10^4^ CFU g^−1^) PGPM-treated plants’ soil (Table 4). The fungal population under control conditions ranged from 18.25 to 8.73 × 10^4^ CFU g^−1^ soil under 0 to 21 DWD, and the PGPM consortium ranged from 19.58 to 12.54 × 10^4^ CFU g^−1^ in soil under 0 to 21 DWD (Table 5). The actinomycetes population ranged from 19.63 to 12.49 × 10^4^ CFU g^−1^ soil under control conditions, under 0 to 21 DWD, and the PGPM consortium ranged from 21.88 to 14.36 × 10^4^ CFU g^−1^ soil under 0 to 21 DWD (Table 6). The maximum bacterial population was recorded under control conditions in the Kashi Abhiman (23.81 × 10^5^ CFU g^−1^ soil) plant’s soil, while it was lowest at 16.94 × 10^5^ CFU g^−1^ soil after 21 DWD in Kashi Aman plants. In PGPM-treated plants’ soil, the highest bacterial population was noted under the control condition in Kashi Abhiman and Kashi Amrit plants’ soil, with respective values of 24.63 × 10^5^ CFU g^−1^ soil in each. Although, we noted the lowest value (17.75 × 10^5^ CFU g^−1^) after 21 DWD in the Kashi Vishesh plant’s soil (Table 6). Reductions in both fungal and actinomycetes populations were also recorded with the increase in DWD in both control and PGPM-treated Kashi Vishesh, Kashi Aman, Kashi Abhiman, and Kashi Amrit plants’ soil. In the case of control plants under 0 DWD, minimums of 10.91 × 10^4^ CFU g^−1^ fungal and 16.13 × 10^4^ CFU g^−1^ actinomycetes populations were recorded in Kashi Vishesh and Kashi Aman plants’ soil, respectively, and a maximum of 19.63 × 10^4^ CFU g^−1^ in the Kashi Amrit plant. Similarly, after 21 DWD, a minimum of 18.25 × 10^4^ CFU g^−1^ was found in Kashi Vishesh and a maximum of 19.63 × 10^4^ CFU g^−1^ soil in Kashi Amrit. In the case of PGPM-treated plants under 0 DWD, minimum and maximum fungal (19.58 × 10^4^ CFU g^−1^) and actinomycetes (21.88 × 10^4^ CFU g^−1^) populations were recorded in the Kashi Amrit plant’s soil. Similarly, after 21 DWD, a maximum (18.23 × 10^4^ CFU g^−1^ soil) and minimum (14.36 × 10^4^ CFU g^−1^) were noted in the Kashi Vishesh plant’s soil (Table 5 and Table 6).

### 3.8. Soil NPK Availability

After 7, 14, and 21 DWD, decreased availability of nitrogen (N), phosphorus (P), and potassium (K) was noted in both control and PGPM-treated Kashi Vishesh, Kashi Aman, Kashi Abhiman, and Kashi Amrit plants’ soil. The maximum nitrogen level was noted at 85.31 (kg/Ha) in the Kashi Amrit plant’s soil after 21 DWD, while the nitrogen level was lowest in the Kashi Vishesh (76.99 Kg/Ha) plant’s soil under 0 DWD.Similarly, when plants were treated with PGPM, the minimum N content was noted in the Kashi Vishesh plant’s soil, 104.28 (Kg/Ha) under 0 DWD, and the maximum, 122.7 (Kg/Ha), in the Kashi Amrit plant’s soil after 21 DWD (Table 7). Similarly, P and K levels were minimum in Kashi Vishesh plant’s soil under 0 DWD in both control and PGPM-treated plants, while P and K were observed maximum in Kashi Amrit plant’s soil after 21 DWD (Table 8 and Table 9).

### 3.9. Soil Enzyme Dehydrogenase, Phosphatase, and Urease Activities

Dehydrogenase, phosphatase, and urease activities were noted in Kashi Vishesh, Kashi Aman, Kashi Abhiman, and Kashi Amrit plants’ soil under 0, 7, 14, and 21 DWD, in both control and PGPM-treated conditions. Dehydrogenase activity was maximum in Kashi Abhiman plant’s soil under 0 DWD, with the respective value of 34.17 (mg TPF·g^−1^ soil·h^−1^) in the control plant’s soil. In PGPM-treated plants’ soil, the maximum was noted in Kashi Amrit plants, with a respective value of 38.33 mg TPF·g^−1^ soil·h^−1^. Similarly, it was lowest after 21 DWD in the Kashi Vishesh plant’s soil in the case of the control, while in PGPM-treated plants, it was noted lowest, 27.30 (mg TPF·g^−1^ soil·h^−1^), in Kashi Vishesh plants (Table 10).

Phosphatase activity with a maximum value of 184.83 (mg pNP·g^−1^ dwt·h^−1^) was recorded in the Kashi Aman plant’s soil in control conditions, and 226.41 (mg pNP·g^−1^ dwt ·h^−1^) in Kashi Aman in the case of PGPM-treated plants’ soil under 0 DWD. A minimum value was observed after 21 DWD (138.76 mg pNP·g^−1^ dwt·h^−1^) in Kashi Amrit plants in the case of control plants’ soil, and 162.50 (mg pNP·g^−1^ dwt·h^−1^) in Kashi Vishesh in the case of PGPM-treated plants. Similarly, a reduction in urease concentration was noted in both PGPM-treated and control plants under 7, 14, and 21 DWD (Table 11).

Regarding the activity of urease measured in Kashi Vishesh, Kashi Aman, Kashi Abhiman, and Kashi Amrit plants’ soil under 0, 7, 14, and 21 DWD, urease activity decreased in both PGPM-treated and control plants, with an increase in DWD. The maximum urease activity of 71.10 (mg NH4-N·g^−1^ dwt·2 h^−1^) was noted in the Kashi Amrit plant’s soil under 0 DWD control plants, while the lowest, at 38.29 (mg NH4-N·g^−1^ dwt·2 h^−1^), was noted in the Kashi Vishesh plant’s soil after 21 DWD. In PGPM-treated plants, the maximum urease activity of 76.55 (mg NH4-N·g^−1^ dwt·2 h^−1^) was noted in the Kashi Amrit plant’s soil under 0 DWD, while the lowest, at 40.32 (mg NH4-N·g^−1^ dwt·2 h^−1^), was noted in the Kashi Vishesh plant’s soil after 21 DWD (Table 12).

### 3.10. Correlation Among the Studied Parameters

**Figure 10:**
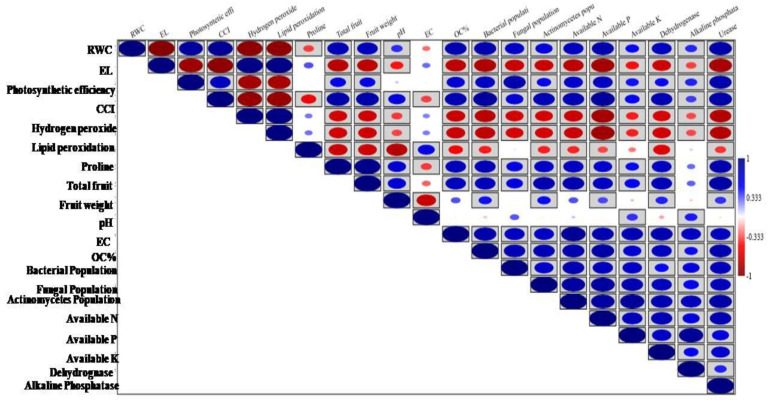
Correlation analysis among the studied parameters. Blue in the box indicates significantly positive correlations, while red in the box indicates significantly negative correlations.

A correlation study among the parameters was performed to study their relationship. Most of the studied parameters showed significant correlation, either positive or negative. The RWC showed a significantly positive correlation with all the parameters, except EL, hydrogen peroxide, lipid peroxidation, and proline. EL showed a significantly negative correlation with all the parameters, except hydrogen peroxide, lipid peroxidation, proline, and EC (Figure 10). Similarly, EC, OC%, bacterial, fungal, and actinomycetes populations, available N, P, and K, and dehydrogenase and alkaline phosphatase levels were significantly positively correlated with OC%, bacterial, fungal, and actinomycetes populations, available N, P, and K, and dehydrogenase and alkaline urease levels.

## 4. Discussion

Among the abiotic stresses, drought is the most destructive and frequent environmental condition that decreases the production and productivity of crops worldwide, consequently negatively affecting plants’ physiological–biochemical yield as well as soil physiochemical properties [21,35]. Approaches such as breeding and genetic engineering could be the most reliable methodologies for increasing crop production and productivity under biotic and abiotic stress conditions. However, the application of PGPMs for drought stress tolerance in crop plants was found to be efficient and effective [36].

Many research reports showed that the application of PGPMs improved the plant growth and yield, along with soil fertility, via direct or indirect mechanisms under abiotic stresses [37]. The alleviation of drought stress in maize, mung bean, chickpea, common bean, and tomato, respectively, by *P. fluorescens*, *P. aeruginosa*, *P. putida*, *Paenibacillus polymixa*, *B. megaterium*, and *T. harzianum*, was reported earlier [38,39].

The present study was executed to observe the changes in the performance of tomato varieties under different levels of drought stress conditions, treated with a hexa-PGPM consortium. The PGPM strains used were *Bacillus megaterium* BHUPSB14, *Pseudomonas fluorescens* BHUPSB06, *Pseudomonas aeruginosa* BHUPSB01, *Pseudomonas putida* BHUPSB0, *Paenibacillus polymixa* BHUPSB17, and *Trichoderma harzianum*, which have effective plant growth promotion properties, such as IAA production, phosphate solubilization, ammonia production, bio-control properties, and siderophore and HCN production [2]. The present study showed the hexa-PGPM consortium’s positive role in the regulation of drought stress tolerance. The hexa-PGPM consortium significantly improved the drought stress tolerance in Kashi Aman, Kashi Abhiman, Kashi Amrit, and Kashi Vishesh varieties. Drought stress adversely affected RWC and EL, which are the physiological parameters frequently used to measure water stress status, as they are directly related to water uptake by the roots and water lost by transpiration. Under drought stress, reduced water and ion uptake by the roots results in low RWC and high EL. In the present study, the application of the hexa-PGPM consortium significantly increased the RWC and reduced EL in the tomato varieties, compared to their control counterparts, under different DWD conditions, which is similar to the results reported in maize and groundnut by using different species of *Pseudomonas* [40,41]. This increase in RWC and decrease in EL may be due to soil aggregation, higher root lengths, and mineral uptake and root colonization by the PGPM consortium [14,19].

The CCI is an important parameter measured under drought stress, as drought causes peroxidation of chlorophyll, reducing its contents. The hexa-PGPM consortium used to inoculate Kashi Vishesh, Kashi Aman, Kashi Abhiman, and Kashi Amrit tomato plants under all DWD conditions showed a significantly higher CCI. The higher CCI in the PGPM-treated plants was reportedly due to the higher uptake of nitrogen under abiotic stress facilitated by the hexa-PGPM consortium. Higher CCI enhances the plant photosynthesis and, ultimately, plant growth and development under stress conditions [42].

Drought-stress-induced osmotic stress decreased the photosynthetic efficiency due to the production of reactive oxygen species (ROS), which destroys the photosynthetic pigments. The hexa-PGPM- inoculated Kashi Vishesh, Kashi Aman, Kashi Abhiman, and Kashi Amrit tomato plants under 0, 7, 14, and 21 DWD conditions exhibited higher photosynthesis efficiency than their control counterparts. Similar findings were also found in rice inoculated with *Trichoderma harzianum* [43].

Under drought stress, the high level of MDA accumulation is the result of peroxidation of membranes, which leads to the damage of the cell membrane. The increased MDA level in the leaf is most commonly analyzed as an oxidative stress indicator responsible for cellular-level damage [44]. In the present study, we found a lower level of MDA accumulation in the hexa-PGPM treated Kashi Vishesh, Kashi Aman, Kashi Abhiman, and Kashi Amrit tomato plants, as compared to the controls, under 0, 7, 14, and 21 DWD stress. Similar results were found in tomato inoculated by *Bacillus cereus* AR156, and in maize by *Pseudomonas putida* and rice by *P. fluorescens*, *P. jessenii*, and *P. Synxantha* [45,46,47]. The H_2_O_2_ level increased under drought stress and could also react with superoxide radicals to produce hydroxyl radicals in plant cells, which can cause membrane lipid peroxidation and protein destruction and, ultimately, death of the cells. In our study, under 0, 7, 14, and 21 DWD stress, hexa-PGPM-treated Kashi Vishesh, Kashi Aman, Kashi Abhiman, and Kashi Amrit tomato plants showed significantly lower levels of H_2_O_2_.This may be due to the high levels of the ROS scavenging agent, which regulated the H_2_O_2_ level in hexa-PGPM-treated plants. Similar to our results, Gusain et al. [47] and Moslemi et al. [48] reported reduced H_2_O_2_ levels due to the application of PGPM in maize and rice, respectively. Under abiotic stresses, proline is produced through protein hydrolysis in plants to compensate for the severity of stress. Under drought stress, proline plays a multifunctional role, such as lipid peroxidation reduction by ROS scavenging, adjustment of cytosolic acidity, and protein and membrane stabilization. The hexa-PGPM-treated Kashi Vishesh, Kashi Aman, Kashi Abhiman, and Kashi Amrit plants showed enhanced proline levels under different DWD stresses, compared to their control counterparts. Elevated proline levels were observed in mung bean by *P. aeruginosa* GGRJ21 [16] and in maize by *P. fluorescens* [38].

Drought stress potentially reduces the crop yield and is the greatest yield-limiting factor in the current scenario, which affects agricultural productivity. The application of the hexa- PGPM consortium in Kashi Vishesh, Kashi Aman, Kashi Abhiman, and Kashi Amrit tomato plants increased the yield in both controls and stressed plants by increasing the number of fruits and the fruits’ weights under different DWD stresses, which might be attributed to the suppression of drought-stress-induced cellular damage and growth promotion by a different mode of action of the hexa-PGPM consortium. Similar results were found in tomato and pea (*Pisum sativum*), respectively [49,50].

Under natural conditions, plant roots secrete root exudates consisting of an organic compound, such as amino acids, sugars, organic acids, fatty acids, putrescine, nucleotides, and vitamins. All these constitute about 6% to 21% of total fixed carbon by plants and act as attractants or nutrients for the PGPM [51]. On the other hand, the application of PGPM improves the soil biological, physical, and chemical properties via a different mode of action, as well as plants’ growth promotion and resistance by releasing phytohormones or small molecules and volatile compounds [52]. The basic soil properties, such as pH, EC, and organic carbon, improved with PGPM treatment under 0, 7, 14, and 21 DWD conditions. There was no significant change in pH recorded in PGPM-treated plants, but EC and OC were significantly improved. We recorded an increase in soil EC with the increase in DWD, but the increment was higher in PGPM-treated plants in all the treatments, and the soil OC % increased with PGPM treatments. However, with the increase in DWD, the soil OC % reduced, and the reduction in PGPM-treated plants was lower compared to their control counterparts. Maintaining soils’ basic properties under drought conditions facilitated by PGPM is the basis for drought stress tolerance. Our findings support those in [37], in which they induced drought tolerance in *Zea mays* with inoculation of *Bacillus amyloliquefaciens* HYD-B17, *B. licheniformis* HYTAPB18, *B. thurengiensis* HYDGRFB19, *Paenibacillus flavisporus* BKB30, and *B. subtilis* RMPB44.

The drought stress negatively affected the soil microbial population (bacterial, fungal, and actinomycetes) and soil enzymes, such as dehydrogenase, urease, and phosphatase. This decrease was due to soil desiccation and lower microbial respiration. In our study, we also reported a decrease in microbial populations under drought stress with an increase in DWD; however, a higher decrease in the fungal population was reported than in bacterial and actinomycetes populations, but the decrease was lower in all PGPM-treated plants, as compared to the controls. Similarly, we also reported a decrease in dehydrogenase, urease, and phosphatase enzyme activities. Our results are similar to the findings in [53,54], which ameliorated drought stress via PGPM application in tomato and maize, respectively. Plants require a total of 17 mineral nutrients, out of which 14 are utilized in inorganic form for normal growth and development by the plants’ roots, but most of the nutrients are not available for uptake by the roots in soil, as they remain in bound form with soil organic or inorganic components or insoluble precipitates, and the PGPM increases the availability of different nutrients via different mechanisms [55]. The drought stress potentially restricts the availability and uptake of nutrients by the plants. In our study, we reported that the inoculation of *Bacillus megaterium* BHUPSB14, *Pseudomonas fluorescens* BHUPSB06, *Pseudomonas aeruginosa* BHUPSB01, *Pseudomonas putida* BHUPSB0, *Paenibacillus polymixa* BHUPSB17, and *Trichoderma harzianum* enhanced the plant nutrients and N, P, K availability under different DWD. This may be due to the maintenance of rhizospheric soil moisture by the colonized PGPMs and other PGPM-induced physico-biochemical processes, which facilitate the nutrient availability under drought stress [55].

## 5. Conclusions

The present study signified the importance of applying PGPMs to enhance drought tolerance and yield in tomato varieties facing progressive drought stress. Inoculating the hexa-PGPM consortium, which included *Bacillus megaterium* BHUPSB14, *Pseudomonas fluorescens* BHUPSB06, *Pseudomonas aeruginosa* BHUPSB01, *Pseudomonas putida* BHUPSB0, *Paenibacillu spolymyxa* BHUPSB17, *Trichoderma harzianum*, *Fusarium oxysporum*, and *Rhizoctonia solani*, significantly improved the CCI, RWC, proline levels, and photosynthetic efficiency, while reducing electrolyte leakage (EL), MDA, and H_2_O_2_ levels in Kashi Vishesh, Kashi Aman, Kashi Abhiman, and Kashi Amrit tomato plants under drought stress. Additionally, the application of these PGPMs improved the soil physicochemical properties, such as pH, EC, OC, and microbial populations, under drought conditions. Treated plants showed increased enzyme activity, including dehydrogenase, phosphatase, and urease, along with greater availability of nitrogen, phosphorus, and potassium (N, P, and K) under drought stress. Notably, the PGPM treatment resulted in higher yields compared to untreated plants under drought conditions. Furthermore, conducting PGPM consortium studies in field conditions is crucial to assess their effectiveness within natural ecosystems that include the entire soil food cycle.

## Figures and Tables

**Table 1:** Effect of PGPM inoculation on tomato plants’ soil pH under drought conditions.

Treatments	Varieties	pH			
		0 Days	7 Days	14 Days	21 Days
Control	Kashi Vishesh	7.99 ± 0.34 ^a^	7.92 ± 0.66 ^a^	7.89 ± 0.23 ^a^	7.88 ± 0.92 ^a^
	Kashi Aman	7.93 ± 0.43 ^a^	7.90 ± 0.43 ^a^	7.87 ± 0.21 ^a^	7.84 ± 0.43 ^a^
	Kashi Abhiman	7.91 ± 0.48 ^a^	7.90 ± 0.33 ^a^	7.88 ± 0.29 ^a^	7.81 ± 0.31 ^ab^
	Kashi Amrit	7.93 ± 0.32 ^a^	7.90 ± 0.41 ^a^	7.87 ± 0.39 ^a^	7.86 ± 0.29 ^a^
Treatment (PGPM)	Kashi Vishesh	7.92 ± 0.63 ^a^	7.89 ± 0.63 ^a^	7.88 ± 0.73 ^a^	7.87 ± 0.49 ^a^
	Kashi Aman	7.89 ± 0.22 ^a^	7.86 ± 0.62 ^a^	7.85 ± 0.66 ^a^	7.82 ± 0.43 ^ab^
	Kashi Abhiman	7.90 ± 0.21 ^a^	7.88 ± 0.31 ^a^	7.86 ± 0.55 ^a^	7.81 ± 0.19 ^ab^
	Kashi Amrit	7.93 ± 0.41 ^a^	7.90 ± 0.64 ^a^	7.87 ± 0.33 ^a^	7.86 ± 0.33 ^a^

Note: Values are means ± SD (standard deviation). Mean values in each column with the same superscript(s) did not differ significantly, but those with different superscripts were significantly different between each treatment, using Duncan’s post hoc multiple comparison tests (*p* ≤ 0.05).

**Table 2:** Effect of PGPM inoculation on tomato plants’ soil electrical conductivity (EC) under drought conditions.

Treatments	Varieties	EC (dSm^−1^)			
		0 Days	7 Days	14 Days	21 Days
Control	Kashi Vishesh	0.448 ± 0.021 ^bc^	0.458 ± 0.043 ^c^	0.467 ± 0.022 ^d^	0.492 ± 0.033 ^d^
	Kashi Aman	0.459 ± 0.029 ^b^	0.480 ± 0.022 ^bc^	0.483 ± 0.033 ^c^	0.503 ± 0.048 ^c^
	Kashi Abhiman	0.464 ± 0.011 ^b^	0.467 ± 0.043 ^c^	0.488 ± 0.039 ^c^	0.506 ± 0.051 ^bc^
	Kashi Amrit	0.467 ± 0.021 ^b^	0.488 ± 0.033 ^b^	0.503 ± 0.044 ^b^	0.512 ± 0.033 ^b^
Treatment (PGPM)	Kashi Vishesh	0.480 ± 0.029 ^b^	0.492 ± 0.043 ^b^	0.506 ± 0.044 ^b^	0.536 ± 0.038 ^a^
	Kashi Aman	0.503 ± 0.031 ^a^	0.506 ± 0.022 ^ab^	0.527 ± 0.039 ^a^	0.527 ± 0.043 ^ab^
	Kashi Abhiman	0.488 ± 0.022 ^ab^	0.512 ± 0.019 ^a^	0.523 ± 0.047 ^a^	0.537 ± 0.054 ^a^
	Kashi Amrit	0.503 ± 0.043 ^a^	0.523 ± 0.034 ^a^	0.537 ± 0.029 ^a^	0.553 ± 0.022 ^a^

Note: Details are mentioned in Table 1.

**Table 3:** Effect of PGPM inoculation on tomato plants’ soil organic carbon (OC) under drought stress conditions.

Treatments	Varieties	OC (%)			
		0 Days	7 Days	14 Days	21 Days
Control	Kashi Vishesh	0.567 ± 0.033 ^c^	0.549 ± 0.029 ^c^	0.498 ± 0.021 ^d^	0.455 ± 0.022 ^d^
	Kashi Aman	0.621 ± 0.021 ^ab^	0.548 ± 0.043 ^c^	0.496 ± 0.023 ^d^	0.485 ± 0.031 ^c^
	Kashi Abhiman	0.601 ± 0.038 ^b^	0.572 ± 0.049 ^b^	0.558 ± 0.033 ^b^	0.524 ± 0.029 ^b^
	Kashi Amrit	0.600 ± 0.022 ^b^	0.553 ± 0.028 ^c^	0.520 ± 0.022 ^c^	0.513 ± 0.033 ^b^
Treatment (PGPM)	Kashi Vishesh	0.591 ± 0.063 ^b^	0.564 ± 0.033 ^c^	0.548 ± 0.053 ^bc^	0.530 ± 0.031 ^ab^
	Kashi Aman	0.653 ± 0.038 ^a^	0.633 ± 0.049 ^a^	0.591 ± 0.039 ^ab^	0.548 ± 0.043 ^a^
	Kashi Abhiman	0.669 ± 0.037 ^a^	0.633 ± 0.033 ^a^	0.601 ± 0.037 ^a^	0.558 ± 0.052 ^a^
	Kashi Amrit	0.659 ± 0.044 ^a^	0.609 ± 0.022 ^ab^	0.594 ± 0.033 ^ab^	0.517 ± 0.021 ^b^

Note: Details are mentioned in Table 1.

**Table 4:** Effect of PGPM inoculation on bacterial populations in soil under drought conditions.

Treatments	Varieties	Bacterial Population (Bacteria (×10^5^ CFU g^−1^ Soil))			
		0 Days	7 Days	14 Days	21 Days
Control	Kashi Vishesh	20.78 ± 1.22 ^b^	19.44 ± 0.88 ^b^	16.94 ± 0.76 ^c^	16.69 ± 066 ^c^
	Kashi Aman	21.84 ± 2.12 ^ab^	20.21 ± 0.75 ^b^	17.75 ± 0.98 ^bc^	15.21 ± 1.24 ^d^
	Kashi Abhiman	23.81 ± 1.05 ^a^	21.51 ± 1.14 ^ab^	21.35 ± 1.45 ^a^	18.90 ± 0.89 ^ab^
	Kashi Amrit	23.17 ± 1.35 ^a^	22.03 ± 1.53 ^a^	21.25 ± 1.02 ^a^	20.60 ± 1.11 ^a^
Treatment (PGPM)	Kashi Vishesh	21.84 ± 1.12 ^ab^	21.51 ± 1.09 ^ab^	19.77 ± 1.54 ^b^	17.75 ± 1.33 ^c^
	Kashi Aman	22.63 ± 1.29 ^a^	20.38 ± 1.34 ^b^	18.90 ± 1.14 ^b^	18.67 ± 0.98 ^ab^
	Kashi Abhiman	24.63 ± 1.43 ^a^	22.93 ± 1.88 ^a^	21.84 ± 1.54 ^a^	19.44 ± 1.26 ^a^
	Kashi Amrit	24.63 ± 1.08 ^a^	23.81 ± 1.54 ^a^	22.63 ± 1.38 ^a^	19.57 ± 1.39 ^a^

Note: Details are mentioned in Table 1.

**Table 5:** Effect of PGPM inoculation on fungal populations in soil under drought conditions.

Treatments	Varieties	Fungal Population (Fungi (×10^4^ CFU g^−1^ Soil))			
		0 Days	7 Days	14 Days	21 Days
Control	Kashi Vishesh	10.91 ± 0.82 ^d^	10.05 ± 0.66 ^c^	8.73 ± 0.32 ^d^	8.30 ± 0.43 ^c^
	Kashi Aman	14.34 ± 0.61 ^c^	13.26 ± 0.53 ^b^	11.64 ± 0.55 ^c^	11.10 ± 0.52 ^bc^
	Kashi Abhiman	15.42 ± 0.54 ^bc^	14.35 ± 0.73 ^b^	13.80 ± 0.85 ^b^	12.17 ± 0.65 ^b^
	Kashi Amrit	18.25 ± 0.77 ^a^	16.70 ± 0.44 ^a^	16.70 ± 1.45 ^a^	15.23 ± 0.62 ^a^
Treatment (PGPM)	Kashi Vishesh	13.65 ± 0.55 ^c^	13.27 ± 0.63 ^b^	12.72 ± 0.54 ^c^	12.54 ± 0.26 ^b^
	Kashi Aman	17.06 ± 1.24 ^ab^	16.20 ± 0.98 ^a^	15.34 ± 1.22 ^b^	14.87 ± 0.54 ^a^
	Kashi Abhiman	18.67 ± 1.12 ^a^	17.21 ± 1.22 ^a^	17.03 ± 1.25 ^a^	14.32 ± 0.73 ^a^
	Kashi Amrit	19.58 ± 1.35 ^a^	16.68 ± 0.65 ^a^	16.68 ± 1.19 ^a^	16.19 ± 1.19 ^a^

Note: Details are mentioned in Table 1.

**Table 6:** Effect of PGPM inoculation on *Actinomycetes* populations in soil under drought stress conditions.

Treatments	Varieties	*Actinomycetes* Population (*Actinomycetes* (Fungi (×10^4^ CFU g^−1^ Soil)))			
		0 Days	7 Days	14 Days	21 Days
Control	Kashi Vishesh	18.35 ± 1.29 ^b^	16.46 ± 0.65 ^c^	16.31 ± 0.98 ^b^	12.49 ± 0.35 ^c^
	Kashi Aman	16.13 ± 0.98 ^c^	14.89 ± 0.72 ^c^	14.20 ± 0.65 ^c^	13.17 ± 0.59 ^c^
	Kashi Abhiman	16.97 ± 0.72 ^c^	15.22 ± 0.65 ^c^	14.85 ± 0.76 ^c^	13.39 ± 0.49 ^c^
	Kashi Amrit	19.63 ± 0.65 ^ab^	18.66 ± 1.25 ^b^	17.39 ± 1.33 ^ab^	16.31 ± 1.02 ^ab^
Treatment (PGPM)	Kashi Vishesh	20.22 ± 1.65 ^a^	18.12 ± 1.22 ^b^	17.04 ± 1.08 ^ab^	14.36 ± 0.88 ^c^
	Kashi Aman	19.32 ± 1.24 ^ab^	18.37 ± 1.32 ^b^	16.57 ± 1.23 ^b^	14.88 ± 0.55 ^bc^
	Kashi Abhiman	19.28 ± 1.22 ^ab^	18.34 ± 1.12 ^b^	17.44 ± 0.92 ^ab^	15.82 ± 0.76 ^b^
	Kashi Amrit	21.88 ± 1.34 ^a^	20.42 ± 1.35 ^a^	19.25 ± 1.22 ^a^	18.23 ± 1.24 ^a^

Note: Details are mentioned in Table 1.

**Table 7:** Effect of PGPM inoculation on nitrogen availability in soil under drought conditions.

Treatments	Varieties	Available Nitrogen (Kg/Ha)			
		0 Days	7 Days	14 Days	21 Days
Control	Kashi Vishesh	94.12 ± 3.68 ^c^	87.20 ± 8.17 ^c^	82.61 ± 4.44 ^c^	76.99 ± 3.68 ^c^
	Kashi Aman	102.41 ± 7.72 ^b^	96.21 ± 7.23 ^b^	89.63 ± 7.32 ^bc^	77.78 ± 4.55 ^c^
	Kashi Abhiman	99.07 ± 8.12 ^b^	91.79 ± 6.66 ^c^	86.95 ± 8.11 ^c^	81.04 ± 7.23 ^bc^
	Kashi Amrit	104.28 ± 7.25 ^b^	96.63 ± 5.88 ^b^	91.53 ± 7.55 ^b^	85.31 ± 6.32 ^b^
Treatment (PGPM)	Kashi Vishesh	104.6 ± 5.55 ^b^	96.9 ± 7.32 ^b^	91.8 ± 8.64 ^b^	85.5 ± 3.88 ^b^
	Kashi Aman	116.4 ± 8.28 ^a^	109.3 ± 8.12 ^a^	101.9 ± 4.65 ^a^	88.4 ± 8.71 ^b^
	Kashi Abhiman	115.2 ± 9.12 ^a^	106.7 ± 7.73 ^a^	101.1 ± 8.62 ^a^	94.2 ± 8.32 ^ab^
	Kashi Amrit	122.7 ± 8.24 ^a^	113.7 ± 8.23 ^a^	107.7 ± 7.35 ^a^	100.4 ± 7.35 ^a^

Note: Details are mentioned in Table 1.

**Table 8:** Effect of PGPM inoculation on phosphorus availability in soil under drought conditions.

Treatments	Varieties	Available Phosphorus (Kg/Ha)			
		0 Days	7 Days	14 Days	21 Days
Control	Kashi Vishesh	46.93 ± 2.22 ^bc^	46.02 ± 3.65 ^c^	40.90 ± 2.54 ^c^	29.50 ± 1.76 ^d^
	Kashi Aman	49.52 ± 1.75 ^b^	45.88 ± 3.66 ^c^	42.52 ± 3.33 ^bc^	38.66 ± 2.22 ^bc^
	Kashi Abhiman	46.83 ± 3.88 ^bc^	46.54 ± 4.22 ^bc^	43.36 ± 2.38 ^bc^	39.81 ± 2.54 ^bc^
	Kashi Amrit	51.76 ± 4.94 ^ab^	49.83 ± 3.54 ^ab^	45.68 ± 3.44 ^b^	44.96 ± 3.21 ^ab^
Treatment (PGPM)	Kashi Vishesh	52.80 ± 3.25 ^a^	49.69 ± 4.24 ^ab^	46.24 ± 3.46 ^ab^	33.08 ± 2.14 ^c^
	Kashi Aman	54.71 ± 2.26 ^a^	48.61 ± 2.88 ^b^	43.51 ± 3.54 ^bc^	44.89 ± 2.98 ^ab^
	Kashi Abhiman	53.40 ± 3.46 ^a^	52.15 ± 4.98 ^a^	48.14 ± 4.12 ^a^	48.13 ± 3.75 ^a^
	Kashi Amrit	56.54 ± 3.76 ^a^	54.99 ± 3.88 ^a^	50.85 ± 3.44 ^a^	48.66 ± 4.12 ^a^

Note: Details are mentioned in Table 1.

**Table 9:** Effect of PGPM inoculation on potassium availability in soil under drought conditions.

Treatments	Varieties	Available Potassium (Kg/Ha)			
		0 Days	7 Days	14 Days	21 Days
Control	Kashi Vishesh	42.03 ± 3.44 ^c^	38.71 ± 2.89 ^cd^	37.32 ± 2.68 ^cd^	34.12 ± 2.22 ^c^
	Kashi Aman	42.46 ± 3.55 ^c^	39.11 ± 3.45 ^cd^	37.70 ± 3.12 ^cd^	34.47 ± 1.66 ^c^
	Kashi Abhiman	43.77 ± 2.88 ^c^	40.31 ± 2.88 ^c^	38.86 ± 3.44 ^c^	35.54 ± 2.54 ^c^
	Kashi Amrit	44.71 ± 3.88 ^c^	41.12 ± 3.45 ^c^	39.70 ± 2.88 ^c^	36.30 ± 3.12 ^c^
Treatment (PGPM)	Kashi Vishesh	53.89 ± 3.55 ^bc^	49.63 ± 4.44 ^bc^	47.85 ± 3.22 ^bc^	43.75 ± 2.33 ^b^
	Kashi Aman	56.03 ± 4.12 ^b^	51.60 ± 4.54 ^b^	49.75 ± 3.66 ^b^	45.49 ± 3.24 ^b^
	Kashi Abhiman	61.65 ± 5.22 ^a^	56.78 ± 2.35 ^a^	54.74 ± 3.54 ^a^	50.05 ± 4.12 ^a^
	Kashi Amrit	64.80 ± 4.24 ^a^	59.69 ± 3.98 ^a^	57.54 ± 3.36 ^a^	52.61 ± 2.98 ^a^

Note: Details are mentioned in Table 1.

**Table 10:** Effect of PGPM inoculation on the activity of dehydrogenase enzyme in soil under drought conditions.

Treatments	Varieties	Dehydrogenase (μg TPF·g^−1^ soil·h^−1^)			
		0 Days	7 Days	14 Days	21 Days
Control	Kashi Vishesh	32.54 ± 1.88 ^b^	29.97 ± 1.54 ^c^	28.89 ± 1.66 ^ab^	26.42 ± 2.39 ^b^
	Kashi Aman	31.41 ± 2.22 ^c^	31.29 ± 1.88 ^ab^	29.07 ± 1.79 ^ab^	27.30 ± 1.55 ^b^
	Kashi Abhiman	34.17 ± 1.44 ^b^	32.45 ± 2.66 ^ab^	31.33 ± 2.35 ^a^	30.79 ± 2.45 ^a^
	Kashi Amrit	32.70 ± 1.64 ^b^	30.14 ± 2.12 ^c^	28.40 ± 1.88 ^ab^	28.30 ± 2.95 ^ab^
Treatment (PGPM)	Kashi Vishesh	39.87 ± 2.11 ^a^	33.49 ± 1.54 ^a^	32.54 ± 2.38 ^a^	27.30 ± 1.44 ^b^
	Kashi Aman	37.48 ± 2.35 ^a^	33.49 ± 1.48 ^a^	30.98 ± 2.15 ^a^	28.39 ± 2.37 ^ab^
	Kashi Abhiman	35.30 ± 2.55 ^b^	33.73 ± 2.38 ^a^	31.97 ± 1.54 ^a^	31.37 ± 1.43 ^a^
	Kashi Amrit	38.33 ± 2.44 ^a^	35.53 ± 1.65 ^a^	30.53 ± 1.45 ^a^	29.03 ± 1.21 ^a^

Note: Details are mentioned in Table 1.

**Table 11:** Effect of PGPM inoculation on the activity of alkaline phosphatase enzyme in soil under drought conditions.

Treatments	Varieties	Alkaline Phosphatase (μg pNPg^−1^dwt h^−1^)			
		0 Days	7 Days	14 Days	21 Days
Control	Kashi Vishesh	162.62 ± 11.45 ^d^	159.09 ± 8.25 ^e^	158.87 ± 12.66 ^d^	143.15 ± 7.35 ^d^
	Kashi Aman	184.83 ± 13.22 ^c^	169.66 ± 4.33 ^d^	156.07 ± 8.42 ^d^	150.40 ± 9.32 ^d^
	Kashi Abhiman	168.92 ± 8.43 ^d^	161.56 ± 13.77 ^de^	154.02 ± 11.46 ^d^	143.36 ± 8.44 ^d^
	Kashi Amrit	170.18 ± 11.8 ^d^	158.64 ± 6.44 ^e^	145.03 ± 9.35 ^e^	138.76 ± 9.14 ^e^
Treatment (PGPM)	Kashi Vishesh	197.77 ± 7.65 ^c^	190.96 ± 8.43 ^c^	179.77 ± 5.64 ^c^	162.50 ± 5.72 ^c^
	Kashi Aman	226.41 ± 14.37 ^b^	220.14 ± 10.66 ^b^	214.92 ± 13.68 ^b^	202.54 ± 11.35 ^b^
	Kashi Abhiman	261.66 ± 13.66 ^a^	257.87 ± 11.34 ^a^	254.17 ± 17.62 ^a^	248.00 ± 9.88 ^a^
	Kashi Amrit	225.32 ± 10.28 ^b^	222.06 ± 5.43 ^b^	218.87 ± 19.92 ^b^	213.56 ± 17.32 ^b^

Note: Details are mentioned in Table 1.

**Table 12:** Effect of PGPM inoculation on the activity of urease enzyme in soil under drought conditions.

Treatments	Varieties	Urease (μg NH_4_-N g^−1^ dwt 2 h^−1^)			
		0 Days	7 Days	14 Days	21 Days
Control	Kashi Vishesh	54.15 ± 2.87 ^d^	43.96 ± 2.66 ^d^	38.97 ± 3.13 ^e^	38.29 ± 2.44 ^d^
	Kashi Aman	62.23 ± 4.61 ^c^	57.52 ± 3.41 ^b^	46.49 ± 2.23 ^c^	45.96 ± 1.86 ^cd^
	Kashi Abhiman	61.19 ± 5.63 ^c^	59.39 ± 4.88 ^b^	47.51 ± 3.66 ^c^	53.99 ± 3.62 ^ab^
	Kashi Amrit	71.10 ± 5.66 ^ab^	67.46 ± 4.12 ^a^	58.62 ± 4.33 ^a^	38.38 ± 1.65 ^d^
Treatment (PGPM)	Kashi Vishesh	61.52 ± 4.78 ^c^	49.76 ± 2.28 ^c^	43.65 ± 3.46 ^d^	40.32 ± 2.44 ^d^
	Kashi Aman	65.55 ± 4.89 ^b^	60.24 ± 4.72 ^b^	51.25 ± 3.66 ^b^	44.44 ± 1.83 ^cd^
	Kashi Abhiman	65.44 ± 5.63 ^b^	61.38 ± 3.44 ^b^	52.66 ± 3.76 ^b^	47.72 ± 2.34 ^c^
	Kashi Amrit	76.55 ± 6.66 ^a^	71.25 ± 3.79 ^a^	63.44 ± 4.54 ^a^	59.82 ± 4.55 ^a^

Note: Details are mentioned in Table 1.

## Data Availability

All the data are present in the manuscript and it will be available with this open access publication.

## References

[1] Ali M.Y., Sina A.A.I., Khandker S.S., Neesa L., Tanvir E.M., Kabir A., Gan S.H. (2020). Nutritional composition and bioactive compounds in tomatoes and their impact on human health and disease: A review. Foods.

[2] Krishna R., Ansari W.A., Soumia P.S., Yadav A., Jaiswal D.K., Kumar S., Verma J.P. (2022). Biotechnological interventions in tomato (*Solanum lycopersicum*) for drought stress tolerance: Achievements and future prospects. BioTech.

[3] Krishna R., Ansari W.A., Jaiswal D.K., Singh A.K., Prasad R., Verma J.P., Singh M. (2021). Overexpression of AtDREB1 and BcZAT12 genes confers drought tolerance by reducing oxidative stress in double transgenic tomato (*Solanum lycopersicum* L.). Plant Cell Rep..

[4] Krishna R., Ansari W.A., Jaiswal D.K., Singh A.K., Verma J.P., Singh M. (2021). Co-overexpression of AtDREB1A and BcZAT12 increases drought tolerance and fruit production in double transgenic tomato (*Solanum lycopersicum*) plants. Environ. Exp. Bot..

[5] Karkute S.G., Krishna R., Ansari W.A., Singh B., Singh P.M., Singh M., Singh A.K. (2019). Heterologous expression of the AtDREB1A gene in tomato confers tolerance to chilling stress. Biol. Plant..

[6] Nath R., Nath D., Li Q., Chen W., Cui X. (2017). Impact of drought on agriculture in the Indo-Gangetic Plain, India. Adv. Atmos. Sci..

[7] Tiwari S., Lata C., Chauhan P.S., Nautiyal C.S. (2016). Pseudomonas putida attunes morphophysiological, biochemical and molecular responses in *Cicer arietinum* L. during drought stress and recovery. Plant Physiol. Biochem..

[8] Vurukonda S.S.K.P., Vardharajula S., Shrivastava M., SkZ A. (2016). Enhancement of drought stress tolerance in crops by plant growth promoting rhizobacteria. Microbiol. Res..

[9] Selvakumar G., Panneerselvam P., Ganeshamurthy A.N. (2012). Bacterial mediated alleviation of abiotic stress in crops. Bacteria in Agrobiology: Stress Management.

[10] Makhadmeh I., Albalasmeh A.A., Ali M., Thabet S.G., Darabseh W.A., Jaradat S., Alqudah A.M. (2022). Molecular characterization of tomato (*Solanum lycopersicum* L.) accessions under drought stress. Horticulturae.

[11] Vimal S.R., Singh J.S., Arora N.K., Singh S. (2017). Soil-plant-microbe interactions in stressed agriculture management: A review. Pedosphere.

[12] Timmusk S., Nevo E. (2011). Plant root associated biofilms: Perspectives for natural product mining. Bacteria in Agrobiology: Plant Nutrient Management.

[13] Gontia-Mishra I., Sapre S., Deshmukh R., Sikdar S., Tiwari S. (2020). Microbe-mediated drought tolerance in plants: Current developments and future challenges. Plant Microbiomes for Sustainable Agriculture.

[14] Fracasso A., Telò L., Lanfranco L., Bonfante P., Amaducci S. (2020). Physiological beneficial effect of Rhizophagusintraradices inoculation on tomato plant yield under water deficit conditions. Agronomy.

[15] Kaushal M., Wani S.P. (2016). Plant-growth-promoting rhizobacteria: Drought stress alleviators to ameliorate crop production in drylands. Ann. Microbiol..

[16] Sarma R.K., Saikia R. (2014). Alleviation of drought stress in mung bean by strain Pseudomonas aeruginosa GGRJ21. Plant Soil.

[17] Ghorbanpour A., Salimi A., Ghanbary M.A.T., Pirdashti H., Dehestani A. (2018). The effect of Trichoderma harzianum in mitigating low temperature stress in tomato (*Solanum lycopersicum* L.) plants. Sci. Hortic..

[18] Kuscu H., Turhan A., Ozmen N., Aydinol P., Demir A.O. (2014). Optimizing levels of water and nitrogen applied through drip irrigation for yield, quality, and water productivity of processing tomato (*Lycopersicon esculentum* Mill.). Hortic. Environ. Biotechnol..

[19] Mona S.A., Hashem A., Abd_Allah E.F., Alqarawi A.A., Soliman D.W.K., Wirth S., Egamberdieva D. (2017). Increased resistance of drought by Trichoderma harzianum fungal treatment correlates with increased secondary metabolites and proline content. J. Integr. Agric..

[20] Khare N., Goyary D., Singh N.K., Shah P., Rathore M., Anandhan S., Sharma D., Arif M., Ahmed Z. (2010). Transgenic tomato cv. Pusa Uphar expressing a bacterial mannitol-1-phosphate dehydrogenase gene confers abiotic stress tolerance. Plant Cell Tissue Organ Cult. (PCTOC).

[21] Ansari W.A., Atri N., Singh B., Kumar P., Pandey S. (2018). Morpho-physiological and biochemical responses of muskmelon genotypes to different degree of water deficit. Photosynthetica.

[22] Shah K., Kumar R.G., Verma S., Dubey R.S. (2001). Effect of cadmium on lipid peroxidation, superoxide anion generation and activities of antioxidant enzymes in growing rice seedlings. Plant Sci..

[23] Heath R.L., Packer L. (1968). Photoperoxidation in isolated chloroplasts: I. Kinetics and stechiometry of fatty acid peroxidation. Arch. Biochem. Biophys..

[24] Bates L.S., Waldren R.P.A., Teare I.D. (1973). Rapid determination of free proline for water-stress studies. Plant Soil.

[25] Jackson M.L. (1973). Soil Chemical Analysis.

[26] Yan F., Schubert S., Mengel K. (1996). Soil pH changes during legume growth and application of plant material. Biol. Fertil. Soils.

[27] Salam A.K., Desvia Y., Sutanto E., Syam T., Nugroho S.G., Kimura M. (1999). Activities of soil enzymes in different land-use systems in middle terrace areas of lampung province, south Sumatra, Indonesia. Soil Sci. Plant Nutr..

[28] Subbiah B.V., Asija G.L. (1956). A rapid procedure for assessment of available nitrogen in rice soils. Curr. Sci..

[29] Olsen S.R., Cole C.V., Watanabe F.S., Dean L. (1954). Estimation of Available Phosphorus in Soil by Extraction with Sodium Carbonate.

[30] Kirkbright G.F., Sargent M. (1974). Atomic Absorption and Fluorescence Spectroscopy.

[31] Aneja V.P., Nelson D.R., Roelle P.A., Walker J.T., Battye W. (2003). Agricultural ammonia emissions and ammonium concentrations associated with aerosols and precipitation in the southeast United States. J. Geophys. Res. Atmos..

[32] Casida L.E., Klein D.A., Santoro T. (1964). Soil dehydrogenase activity. Soil Sci..

[33] Kandeler E., Gerber H. (1988). Short-term assay of soil urease activity using colorimetric determination of ammonium. Biol. Fertil. Soils.

[34] Eivazi F., Tabatabai M.A. (1977). Phosphatases in soils. Soil Biol. Biochem..

[35] Ansari W.A., Atri N., Ahmad J., Qureshi M.I., Singh B., Kumar R., Rai V., Pandey S. (2019). Drought mediated physiological and molecular changes in muskmelon (*Cucumis melo* L.). PLoS ONE.

[36] Nautiyal C.S., Srivastava S., Chauhan P.S., Seem K., Mishra A., Sopory S.K. (2013). Plant growth-promoting bacteria Bacillus amyloliquefaciens NBRISN13 modulates gene expression profile of leaf and rhizosphere community in rice during salt stress. Plant Physiol. Biochem..

[37] Vardharajula S., Zulfikar Ali S., Grover M., Reddy G., Bandi V. (2011). Drought-tolerant plant growth promoting *Bacillus* spp.: Effect on growth, osmolytes, and antioxidant status of maize under drought stress. J. Plant Interact..

[38] Ansary M.H., Rahmani H.A., Ardakani M.R., Paknejad F., Habibi D., Mafakheri S. (2012). Effect of Pseudomonas fluorescent on proline and phytohormonal status of maize (*Zea mays* L.) under water deficit stress. Ann. Biol. Res..

[39] Silambarasan S., Logeswari P., Cornejo P., Kannan V.R. (2019). Role of plant growth–promoting rhizobacterial consortium in improving the Vigna radiata growth and alleviation of aluminum and drought stresses. Environ. Sci. Pollut. Res..

[40] Fazal A., Bano A. (2016). Role of plant growth-promoting rhizobacteria (PGPR), biochar, and chemical fertilizer under salinity stress. Commun. Soil Sci. Plant Anal..

[41] Ghorai S., Pal K.K., Dey R. (2015). Alleviation of Salinity Stress in Groundnut by Application of PGPR. Int. Res. J. Eng. Technol..

[42] Khambani L.S., Hassen A.I., Regnier T. (2019). Rhizospheric bacteria from pristine grassland have beneficial traits for plant growth promotion in maize (*Zea mays* L.). Cogent Biol..

[43] Shukla N., Awasthi R.P., Rawat L., Kumar J. (2012). Biochemical and physiological responses of rice (*Oryza sativa* L.) as influenced by Trichoderma harzianum under drought stress. Plant Physiol. Biochem..

[44] Lata C., Prasad M. (2011). Role of DREBs in regulation of abiotic stress responses in plants. J. Exp. Bot..

[45] Wang C.J., Yang W., Wang C., Gu C., Niu D.D., Liu H.X., Wang Y.P., Guo J.H. (2012). Induction of drought tolerance in cucumber plants by a consortium of three plant growth-promoting rhizobacterium strains. PLoS ONE.

[46] Habibi D., Moslemi Z., Ardakani M.R., Mohammadi A., Asgharzadeh A. (2010). Effects of super absorbent polymer and plant growth promoting rhizobacteria (PGPR) on yield and oxidative damage of maize under drought stress. Proceedings of the 2010 International Conference on Chemistry and Chemical Engineering.

[47] Gusain Y.S., Singh U.S., Sharma A.K. (2015). Bacterial mediated amelioration of drought stress in drought tolerant and susceptible cultivars of rice (*Oryza sativa* L.). Afr. J. Biotechnol..

[48] Moslemi Z., Habibi D., Asgharzadeh A., Ardakani M.R., Mohammadi A., Sakari A. (2011). Effects of super absorbent polymer and plant growth promoting rhizobacteria on yield and yield components of maize under drought stress and normal conditions. Afr. J. Agric. Res..

[49] Arshad M., Shaharoona B., Mahmood T. (2008). Inoculation with Pseudomonas spp. containing ACC-deaminase partially eliminates the effects of drought stress on growth, yield, and ripening of pea (*Pisum sativum* L.). Pedosphere.

[50] Bakr J., Daood H.G., Pék Z., Helyes L., Posta K. (2017). Yield and quality of mycorrhized processing tomato under water scarcity. Appl. Ecol. Environ. Res..

[51] Lugtenberg B. (2015). Life of microbes in the rhizosphere. Principles of Plant-Microbe Interactions: Microbes for Sustainable Agriculture.

[52] Ortiz-Castro R., López-Bucio J. (2019). Phytostimulation and root architectural responses to quorum-sensing signals and related molecules from rhizobacteria. Plant Sci..

[53] Shintu P.V., Jayaram K.M. (2015). Phosphate solubilising bacteria (*Bacillus polymyxa*)-An effective approach to mitigate drought in tomato (*Lycopersicon esculentum* Mill.). Trop. Plant Res..

[54] Naseem H., Bano A. (2014). Role of plant growth-promoting rhizobacteria and their exopolysaccharide in drought tolerance of maize. J. Plant Interact..

[55] Etesami H., Adl S.M. (2020). Plant growth-promoting rhizobacteria (PGPR) and their action mechanisms in availability of nutrients to plants. Phyto-Microbiome in Stress Regulation. Environmental and Microbial Biotechnology.

